# Neutralizing antibody responses over time in a demographically and clinically diverse cohort of individuals recovered from SARS-CoV-2 acquisition in Africa: A cohort study

**DOI:** 10.1371/journal.pgph.0005156

**Published:** 2025-09-11

**Authors:** Nonhlanhla N. Mkhize, Shuying Sue Li, Jiani Hu, Samuel T. Robinson, Zaheer Hoosain, Nigel Garrett, Zvavahera M. Chirenje, Llewellyn Fleurs, Haajira Kaldine, Tandile Modise, Penny L. Moore, April K. Randhawa, Anton M. Sholukh, Julia Hutter, Laura Polakowski, Lawrence Corey, Katherine Gill, David C. Montefiori, Holly Janes, Shelly Karuna, for the HVTN 405/HPTN 1901 Study Team

**Affiliations:** 1 SAMRC Antibody Immunity Research Unit, University of the Witwatersrand, Johannesburg, South Africa; 2 National Institute for Communicable Diseases, National Health Laboratory Service, Johannesburg, South Africa; 3 Vaccine and Infectious Disease Division, Fred Hutchinson Cancer Research Center, Seattle, Washington, United States of America; 4 Josha Research Centre, Bloemfontein, South Africa; 5 Centre for the AIDS Programme of Research in South Africa (CAPRISA), University of KwaZulu-Natal, Durban, South Africa; 6 Department of Public Health Medicine, School of Nursing and Public Health, University of KwaZulu-Natal, Durban, South Africa; 7 University of Zimbabwe Clinical Trials Research Centre, Harare, Zimbabwe; 8 Department of Obstetrics, Gynecology and Reproductive Science, University of California San Francisco, San Francisco, California, United States of America; 9 Desmond Tutu HIV Centre, University of Cape Town, Cape Town, South Africa; 10 Division of AIDS, National Institute of Allergy and Infectious Diseases, National Institutes of Health, Bethesda, Maryland, United States of America; 11 Department of Surgery, Duke University Medical Center, Durham, North Carolina, United States of America; University of California Irvine, UNITED STATES OF AMERICA

## Abstract

COVID-19 has affected millions worldwide. Research characterized immune responses of individuals who acquired SARS-CoV-2 and identified co-factors, such as HIV, associated with greater likelihood of poor clinical outcomes. SARS-CoV-2-specific neutralizing antibodies (nAbs) are a strong correlate of protection but their elicitation in people living with HIV (PLWH), and particularly in southern Africa, is less well characterized. HVTN 405/HPTN 1901 was an observational cohort study of individuals recently recovered from SARS-CoV-2. We describe 323 participants enrolled early in the pandemic (June 2020 to January 2021) in Zambia (n = 12), Malawi (n = 13), Zimbabwe (n = 59), and South Africa (n = 239), profiling their SARS-CoV-2-specific nAb responses and associations with demographics, comorbidities, disease severity, and time since diagnosis based on linear and logistic regression. Participants’ median age was 39 years, 63.5% were assigned female sex at birth, 71.2% were black African, and 39 (12.1%) were PLWH. Approximately one in four participants (25.7%) had asymptomatic SARS-CoV-2, 47.4% were symptomatic but not hospitalized, and 26.9% were hospitalized with COVID-19. Participants in these groups were enrolled at a median of 51.5 days, 53 days, and 60 days post-SARS-CoV-2 diagnosis, respectively. SARS-CoV-2 nAbs were measured in serum using one of two calibrated assays. Most (291/322, 90.4%) participants had positive nAb responses at enrollment. Across all participants, nAb responses generally declined in magnitude between enrollment and 2–3 months thereafter, then increased through month 12 coincident with epidemiologically observed new waves of acquisition. In a multivariate model adjusted for potentially confounding factors, PLWH had a 65% lower geometric mean (GM) nAb ID50 titer compared to people without HIV (PWOH) (GMR: 0.35, p = 0.003, q = 0.006). Greater disease severity, older age (>55 years), high BMI (≥30) and diabetes were associated with higher nAb ID50 titers (all p < 0.05, all q < 0.20).These findings are important, as nAb titers are predictive of vulnerability to COVID-19.

## Introduction

With over 750 million reported cases of Coronavirus Disease 2019 (COVID-19) worldwide as of November 2024 [1], there remain opportunities to analyze and archive important data on the immune response to the causative agent, Severe Acute Respiratory Syndrome Coronavirus 2 (SARS-CoV-2). Acquisition of SARS-CoV-2 has been shown to manifest across a broad range of clinical severities, ranging from asymptomatic cases to severe disease, which can result in death. Early in the pandemic, characterizing the immune responses of convalescent individuals was a critical step in establishing benchmarks for vaccine-induced immunogenicity, and this remains relevant as vaccines are developed to target emerging viral strains.

SARS-CoV-2-specific neutralizing antibodies (nAbs) have been established as a correlate of vaccine-induced protection against COVID-19 [2,3]. Some natural history studies have also found binding or neutralizing antibodies induced by SARS-CoV-2 acquisition (note that acquisition is being used rather than infection in alignment with National Institute of Allergy and Infectious Diseases guidelines on non-stigmatizing language) to inversely correlate with subsequent reacquisition, although the strength of the findings has varied across studies [3–10]. Numerous studies have identified that prior infection plus vaccination, or “hybrid immunity”, affords the strongest antibody response and the most effective protection [11–14]. Yet antibody responses to both vaccination and to SARS-CoV-2 acquisition are variable; thus, significant efforts have been undertaken to understand predictors of the nAb response and, by extension, immune protection. Comorbidities as well as clinical and demographic factors associated with different severities of COVID-19 are of interest, and several associations have been identified [15,16]. Notably, people living with HIV (PLWH) have been identified as a group with a higher likelihood of more severe outcomes [17–21], and yet comparative studies of their immune responses to acquisition are still limited and varied in their conclusions [22–26].

Approximately half of the estimated 39.9 million PLWH in 2023 resided in eastern and southern Africa [27]. For this and other reasons, the course of the COVID-19 pandemic in Africa has been unique [28–30]. Early reports suggested lower-than-expected rates of incidence and death [31–33], though this was likely influenced by inconsistencies in health infrastructure, including the availability of testing, reporting systems, and other resources, particularly early in the pandemic. This only exacerbated a persistent and pervasive lag of high-quality clinical data emanating from African populations.

Early in the pandemic, the hereon reported HVTN 405/HPTN 1901 trial was established with the goal of characterizing SARS-CoV-2-specific immunity in a multinational cohort of convalescent individuals. Results based on the subset of participants from the Americas (United States and Peru) have been described previously and demonstrated that nAb responses tended to peak 1 month after diagnosis and waned substantially over the following four months, albeit with considerable heterogeneity associated with demographic factors, pre-existing medical co-morbidities, and COVID-19 severity [34]. Peak antibody responses among PLWH with COVID-19 (i.e., symptomatic SARS-CoV-2 acquisition) were diminished compared to antibody responses among their counterparts without HIV (PWOH), and antibody responses among PLWH did not correlate with COVID-19 severity, as they did among PWOH [25].

In this report we considered participants enrolled in HVTN 405/HPTN 1901 in Africa: we characterized their nAb responses to SARS-CoV-2 acquisition and examined how these responses varied with COVID-19 severity, as well as demographic and clinical characteristics, including HIV status.

## Materials and methods

### Trial design

HVTN 405/HPTN 1901 was an observational cohort study (ClinicalTrials.gov NCT04403880) that enrolled 759 participants with recent SARS-CoV-2 acquisition at 53 clinical research sites in Malawi, South Africa, Zambia, Zimbabwe, Peru, and the United States [35]. This manuscript includes neutralizing antibody data for participants that enrolled in the southern Africa sites. Enrollment at these sites occurred between July 29, 2020, and March 31, 2021, and this analysis was further restricted to the 323 participants who were diagnosed prior to October 1, 2020. Written informed consent was obtained from each participant at enrollment using a standardized protocol consent form. Results pertaining to participants from Peru and United States have been reported elsewhere [25,34]. Participants were stratified by COVID-19 symptom severity (asymptomatic, symptomatic outpatient, or hospitalized) and by age (18–55 or >55 years of age). For symptomatic outpatient and hospitalized participants, SARS-CoV-2 diagnosis dates were based on the date of the first positive SARS-CoV-2 nucleic acid amplification test (NAAT) or the date of symptom onset, whichever occurred earlier. Symptomatic outpatient participants and participants hospitalized due to COVID-19 were eligible to enroll 1–8 weeks after disease resolution. Participants who were asymptomatic, despite confirmed SARS-CoV-2 acquisition, defined by a reported positive SARS-CoV-2 test (PCR or antigen), were eligible to enroll 2–10 weeks after diagnosis. Additional information on study inclusion and exclusion criteria is included in S1 Text.

Demographic and clinical data were collected for all participants at a required baseline study visit, and at optional follow-up visits 2, 4, and 12 months after enrollment. Medical history (including HIV and COVID-19 history) was documented by the enrolling clinic using participant health records. Blood and nasopharyngeal samples were collected for virologic and immunologic assays. Ethics Committee or Institutional Review Board (IRB) approval was granted by a Central IRB in the United States (Advarra IRB) and, as applicable, by individual review boards and applicable regulatory agencies for the clinical research sites.

### COVID severity grading

COVID-19 severity was defined using a custom grading scale that followed the Division of AIDS (DAIDS), Food and Drug Administration (FDA), and World Health Organization (WHO) grading scales as closely as possible given the constraints posed by the COVID-19 symptom and treatment data that were collected. From least to most severe, severity at the time of enrollment was based on the following scale: 1: Asymptomatic, 2: Symptomatic, not hospitalized, 3: Hospitalized, no supplemental oxygen, 4: Hospitalized, with supplemental oxygen, but no ICU or intubation, 5: Hospitalized, with ICU or intubation, 6: Hospitalized, with ECMO (extracorporeal membrane oxygenation). Severity categories were mutually exclusive and were assessed from highest severity to lowest. For the purposes of analysis, participants with grades 3–6 were combined to form a single “hospitalized” severity group.

### Neutralizing antibody detection

For PWOH, nAbs against SARS-CoV-2 were measured in 293T/ACE2 cells as a function of reduction in Tat-induced luciferase (Luc) reporter gene expression after a single round of infection with lentivirus particles pseudotyped with the SARS-CoV-2 Wuhan-1/D614G Spike protein [36]. SARS-CoV-2 pseudotyped lentiviruses were prepared by co-transfecting 293T cells with the SARS-CoV-2 Spike plasmid, a firefly luciferase-reporter gene plasmid, a TMPRSS-2-expressing plasmid, and a lentivirus backbone plasmid. Pseudovirions were titrated for infectivity and assayed for neutralization in 293T/ACE2 cells. Luciferase activity was quantified by luminescence and was directly proportional to the number of infectious virus particles present in the test samples.

In PLWH on antiretroviral therapy (ART), the ART interferes with the 293T/ACE2 lentivirus assay. Thus, for PLWH, nAbs were measured by the vesicular stomatitis virus (VSV) pseudovirus neutralization assay described by Sholukh et al (S2 Text) [37]. VSV pseudovirus was prepared using a codon optimized gene of SARS-CoV-2 Spike protein (YP_009724390.1) cloned into a pcDNA3.1 (PsVSVLucD19) and VSV(G*∆G-luciferase) system purchased from Kerafast (Boston, MA) [38,39].

Neutralization titers were defined as the inhibitory dilution of serum samples at which relative light units (RLUs) were reduced by either 50% (ID50) or 80% (ID80) compared to virus control wells. A positive response was defined as neutralization ≥50% at the lowest serum dilution tested (1:10). For negative responses, the ID50 (ID80) titer was set to 5.

### Statistical analysis

Neutralizing antibody responses for the 293T/ACE2 lentivirus and VSV assays were calibrated prior to analysis to facilitate comparisons across PLWH/PWOH groups (S2 and S3 Text, and S1 Fig).

Regression models were used to associate nAb responses at enrollment with individual participant baseline characteristics: COVID-19 severity, age (>55 vs 18–55), body mass index (BMI) (≥30 vs < 30), HIV status, prolonged viral shedding (detection of SARS-CoV-2 on two tests at least 21 days apart), preexisting medical conditions (hypertension; asthma, COPD, or emphysema; and diabetes), smoking history, sex assigned at birth, and days since SARS-CoV-2 diagnosis. Models were adjusted for a pre-specified set of covariates that have been previously found to associate with nAb titers COVID-19 severity, age, sex at birth, African region (RSA vs. non-RSA), and days since SARS-CoV-2 diagnosis. We refer to these as “adjustment factors”. nAb response titers were modeled on the log scale using linear regression, and nAb response positivity was modeled using Firth logistic regression [40].

The Benjamini and Hochberg method [41] of controlling the false discovery rate was used to adjust for multiple comparisons. A false-discovery rate, or q-value, of less than 0.20 was considered statistically significant.

To describe the trajectory of nAb responses over time since SARS-CoV-2 diagnosis by COVID-19 severity and HIV status, a generalized additive mixed model (GAMM) was used [42]; nAb responses were modeled on the log scale. The R mgcv package was used with order 2 of penalty derivative and basis dimension 50.

All analyses were done using R [43].

## Results

### Study population

A total of 323 participants from four countries were enrolled in the African cohort between 29 July 2020 and 18 January 2021, diagnosed prior to 1 October 1, 2020, and included in this analysis (Table 1). The majority of participants were from South Africa (RSA) (n = 239) compared to 84 participants from the other African countries (Zambia (n = 12), Malawi (n = 13), Zimbabwe (n = 59)). Across all participants, more experienced symptomatic COVID-19 without hospitalization (n = 153, 47.4%), compared to those with asymptomatic acquisition (n = 83; 25.7%), or requiring hospitalization (n = 87; 26.9%). The participant pool was diverse: 63.5% were assigned female sex at birth, the median age was 39 years (interquartile range [IQR]: 31–51, range: 18–84), 18.0% were 55 years of age or older, 71.2% were black African, and multiple comorbidities were well represented. Across all severities, 39 (12.1%) participants were PLWH, of which 7 (17.9%) were asymptomatic, 13 (33.3%) were symptomatic but did not require hospitalization, and 19 (48.7%) were hospitalized.

**Table 1:** Participant characteristics at the time of enrollment for the Africa cohort.

	Total (N = 323)	Asymptomatic (N = 83)	Symptomatic, Not Hospitalized (N = 153)	Hospitalized (N = 87)
**Age**				
Mean (SD)	40.9 (13.43)	36.8 (14.43)	41.3 (12.67)	44 (12.92)
Median (IQR)	39 (31, 51)	32 (26, 46.5)	40 (32, 50)	43 (34, 55)
Range	18 - 84	18-76	19-84	20-72
**Age Range, n (%)**				
18 - 55	265 (82.0%)	69 (83.1%)	130 (85%)	66 (75.9%)
> 55	58 (18.0%)	14 (16.9%)	23 (15%)	21 (24.1%)
**Sex Assigned at Birth, n (%)**				
Female	205 (63.5%)	40 (48.2%)	107 (69.9%)	58 (66.7%)
Male	118 (36.5%)	43 (51.8%)	46 (30.1%)	29 (33.3%)
**HIV Status, n (%)**				
People Living with HIV (PLWH)	39 (12.1%)	7 (8.4%)	13 (8.5%)	19 (21.8%)
People Without HIV (PWOH)	284 (87.9%)	76 (91.6%)	140 (91.5%)	68 (78.2%)
**Race**				
White	9 (2.8%)	1 (1.2%)	7 (4.6%)	1 (1.1%)
Black	230 (71.2%)	65 (78.3%)	103 (67.3%)	63 (72.4%)
Asian	10 (3.1%)	1(1.2%)	6 (3.9%)	3 (3.4%)
Other*	72 (22.3%)	16 (19.3%)	36 (23.5%)	20 (23%)
**Ethnicity**				
Hispanic or Latinx	1 (0.3%)	0 (0.0%)	0 (0.0%)	1 (1.1%)
** **Not Hispanic or Latinx	322 (99.7%)	83 (100%)	153 (100%)	86 (98.8%)
**BMI**				
Mean (SD)	30.8 (8.36)	28.7 (8.31)	30.8 (7.84)	32.7 (8.9)
Median (IQR)	29.2 (24.9, 34.9)	26.9 (22.1, 33)	29.4 (25.8, 34.8)	30.4 (26.8, 37.1)
Range	15.2 - 65.1	17 - 55.5	15.2 - 57.9	16.7 - 65.1
**BMI Category, n (%)**				
< 30	171 (52.9%)	53 (63.9%)	78 (51%)	40 (46%)
≥ 30	148 (45.8%)	30 (36.1%)	72 (47.1%)	46 (52.9%)
**Days Since SARS-CoV-2 Diagnosis**				
Mean (SD)	54.1 (20.19)	50.6 (15.36)	51.4 (20.84)	62.1 (21.07)
Median (IQR)	55 (38, 66)	51.5 (38, 62)	53 (34.5, 65)	60 (46.5, 76)
Range	14 - 185	14 - 90	15 - 185	22 - 137
**Days Since SARS-CoV-2 Diagnosis Range Category**				
< 28	24 (7.4%)	5 (6%)	17 (11.1%)	2 (2.3%)
28 - < 42	68 (21.1%)	19 (22.9%)	37 (24.2%)	12 (13.8%)
42 - < 56	70 (21.7%)	23 (27.7%)	24 (15.7%)	23 (26.4%)
56+	158 (48.9%)	35 (42.2%)	73 (47.7%)	50 (57.5%)
**Medical History, n (%)**				
Currently smoke cigarettes or marijuana	31 (9.6%)	10 (12%)	16 (10.5%)	5 (5.7%)
Ever smoked cigarettes or marijuana	62 (19.2%)	19 (22.9%)	33 (21.6%)	10 (11.5%)
Hypertension	77 (23.8%)	13 (15.7%)	30 (19.6%)	34 (39.1%)
Asthma, COPD, or Emphysema	16 (5%)	2 (2.4%)	9 (5.9%)	5 (5.7%)
Diabetes	45 (13.9%)	7 (8.4%)	18 (11.8%)	20 (23%)
Prolonged Viral Shedding	5 (1.5%)	1 (1.2%)	2 (1.3%)	2 (2.3%)

*“Other” Race was an option on the case report form; participants were asked to specify, and responses included the following: African (n = 17), Black African or African Black or SA black (n = 28), Colored (n = 8), Indian or SA Indian (n = 15), Mixed race (n = 2), and Nsundu (n = 1).

Descriptively, COVID-19 severity was associated with several participant characteristics (Table 1). Participants 18–55 years old constituted a larger proportion of those in the symptomatic non-hospitalized category (85.0%) than in the asymptomatic (83.1%) and hospitalized (75.9%) groups, whilst adults >55 constituted a larger portion of the hospitalized (24.1%) group than the asymptomatic (16.9%) and symptomatic non-hospitalized (15.0%) groups. Participants assigned female sex at birth were represented more frequently in the symptomatic non-hospitalized (69.9%) and hospitalized (66.7%) groups compared to the asymptomatic group (48.2%). PLWH (21.8%) and individuals who had hypertension (39.1%) were more frequent in the hospitalized group compared to the other groups (asymptomatic, 8.4% PLWH and 15.7% with hypertension; symptomatic, 8.5% PLWH and 19.6% with hypertension).

Participants who were asymptomatic were enrolled a median of 51.5 days after SARS-CoV-2 acquisition diagnosis (IQR: 38–62 days), while symptomatic, non-hospitalized participants were enrolled a median of 53 days post-diagnosis (IQR: 34.5 to 65 days) and hospitalized participants were enrolled a median of 60 days post-diagnosis (IQR: 46.5 to 76 days).

### Descriptive analysis of neutralizing antibody responses at enrollment

**Fig 1:**
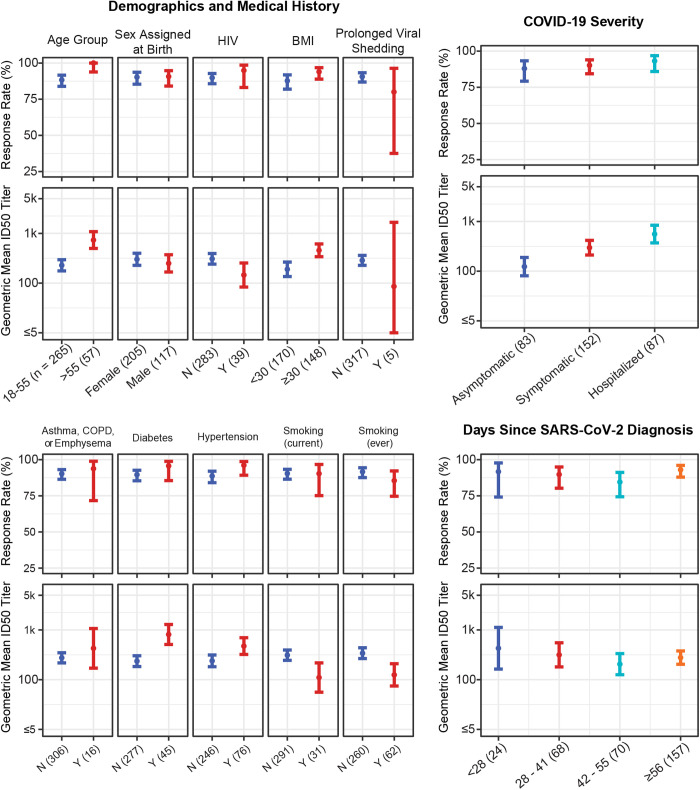
Estimated SARS-CoV-2 neutralizing antibody (nAb) response rate and geometric mean (GM) nAb ID50 titers at enrollment by select baseline participant characteristics. Points show estimated response rates and GM ID50 titers; lines show 95% confidence intervals. Y = yes; N = no. The number of participants in each category is given in parentheses in the labels along the x-axis.

Most participants had positive SARS-CoV-2 nAb responses at enrollment: response rates were 87.95% (95% confidence interval (CI): 79.22%, 93.32%) for asymptomatic participants, 90.13% (95% CI: 84.36%, 93.93%) for symptomatic non-hospitalized participants, and 93.10% (95% CI: 85.76%, 96.80%) for hospitalized participants (S1 Table, Fig 1). Response magnitudes were more variable, with geometric mean (GM) ID50 titers of 123.5 (95% CI: 81.0, 188.4), 296.9 (211.6, 416.7), and 547.5 (95% CI: 362.3, 827.5) in the asymptomatic, symptomatic, and hospitalized groups, respectively. Response rates were high (>80%) across all subgroups of participants defined by baseline participant characteristics (S1 Table, Fig 1). GM ID50 titers ranged from a low of 85.6 (95% CI: 4.4, 1666.8) in the five participants with prolonged viral shedding, (next lowest was 110.5 [95% CI: 56.0, 218.0] among 31 current smokers), to a high of 790.4 (95% CI: 493.9, 1265.0) among 45 participants with diabetes. Numerically, higher nAb titers were observed for individuals >55 years old vs. 18–55 (GM ID50: 719.0 [95%CI: 484.6, 1066.9] vs. 228.0 [95%CI: 175.9, 295.5]), without prolonged viral shedding (GM ID50: 284.7 [95%CI: 226.2, 358.3] vs. 85.6 [95%CI: 4.4, 1666.8]) and with diabetes (ID50: 790.4 [95%CI: 493.9, 1265.0] vs. 236.0 [95%CI: 183.7, 303.1])

### Association between participant characteristics and neutralizing antibody responses at enrollment

Regression analyses revealed numerous factors independently associated with anti-SARS-CoV-2 nAb response positivity and ID50 titer at enrollment (Table 2, Fig 1). After controlling for the adjustment factors, nAb response rates did not differ by COVID-19 severity (p = 0.546, q = 0.728), though nAb titers did: participants who were hospitalized with COVID-19 symptoms exhibited over 4-fold higher geometric mean nAb ID50 titers compared to asymptomatic participants (geometric mean ratio [GMR] = 4.62, 95% CI: 2.51, 8.51; p < 0.001, q < 0.001) and 1.73-fold higher geometric mean nAb titer compared to symptomatic participants who were not hospitalized (95% CI for GMR: 1.01, 2.96; p = 0.046, q = 0.066). Symptomatic participants who were not hospitalized had 2.67-fold higher geometric mean nAb titer compared to asymptomatic participants (95% CI for GMR: 1.56, 4.56; p < 0.001, q = 0.001).

**Table 2:** Results of multivariate models associating baseline participant characteristics with SARS-CoV-2 neutralizing antibody (nAb) ID50 titer among all participants at enrollment. Each predictor of interest was studied independently for its association with a positive nAb response (using Firth logistic regression) and with the nAb ID50 titer (using linear regression). Each regression model adjusts for the following adjustment factors selected based on prior literature: COVID-19 severity, age, sex at birth, African region, and days since SARS-CoV-2 acquisition diagnosis. (OR = Odds Ratio; GMR = Geometric Mean Ratio).

	Positive nAb Response				nAb ID50 titer			
	OR	95% CI	P-Value	Q-Value	GMR	95% CI	P-Value	Q-Value^#^
Age (>55 vs. 18–55)	14.02*	[1.90, 1789.08]	**0.004**	**0.036**	2.91	[1.65, 5.14]	**<0.001**	**0.001**
BMI (≥30 vs < 30)	2.06	[0.85, 5.23]	0.111	0.410	2.12	[1.32, 3.40]	**0.002**	**0.005**
HIV (Yes vs. No)	1.97	[0.56, 10.44]	0.317	0.719	0.35	[0.18, 0.69]	**0.003**	**0.006**
COVID-19 Severity			0.546	0.728			**<0.001**	**<0.001**
Symptomatic (not hospitalized) vs. Asymptomatic	1.59	[0.63, 3.92]	0.316	0.719	2.67	[1.56, 4.56]	**<0.001**	**0.001**
Hospitalized vs. Symptomatic (not hospitalized)	0.99	[0.36, 2.95]	0.989	0.991	1.73	[1.01, 2.96]	**0.046**	**0.066**
Hospitalized vs. Asymptomatic	1.58	[0.53, 4.98]	0.412	0.719	4.62	[2.51, 8.51]	**<0.001**	**<0.001**
Asthma, COPD, or Emphysema (Yes vs. No)	0.93	[0.20, 8.90]	0.933	0.991	1.18	[0.44, 3.19]	0.738	0.814
Diabetes (Yes vs. No)	1.01	[0.29, 5.28]	0.991	0.991	2.17	[1.14, 4.16]	**0.019**	**0.031**
Hypertension (Yes vs. No)	1.57	[0.52, 6.29]	0.449	0.719	1.03	[0.59, 1.83]	0.908	0.908
Cigarettes or Marijuana Smoker (Current) (Yes vs. No)	0.63	[0.19, 2.67]	0.497	0.723	0.34	[0.16, 0.72]	**0.005**	**0.010**
Cigarettes or Marijuana Smoker (Ever) (Yes vs. No)	0.45	[0.18, 1.21]	0.111	0.410	0.34	[0.19, 0.60]	**<0.001**	**0.001**
Days Since SARS-CoV-2 Diagnosis (Per day)	1.02	[1.00, 1.04]	0.128	0.410	1.00	[0.99, 1.01]	0.673	0.814
Sex Assigned at Birth (Male vs. Female)	1.41	[0.62, 3.42]	0.421	0.719	1.07	[0.67, 1.72]	0.763	0.814
Race (Non-Black vs. Black)	0.87	[0.36, 2.28]	0.766	0.942	0.86	[0.53, 1.41]	0.560	0.747

# Q-value is the false discovery rate (FDR)-adjusted p-value for multiple comparisons.

*Large odds ratio and uncertainty thereof is due to the observation of a 100% response rate for those >55 years of age.

Too few participants exhibited prolonged viral shedding to make formal comparisons of this characteristic.

After controlling for the adjustment factors, participants who had ever been cigarette or marijuana smokers, or were currently cigarette or marijuana smokers, had lower anti-SARS-CoV-2 GM ID50 titer at enrollment (GMR = 0.34, 95% CI: 0.16, 0.72; p = 0.005, q = 0.010 for current smokers; GMR = 0.34, 95% CI: 0.19, 0.60, p < 0.001, q = 0.001 for ever smokers). Older participants (age > 55 vs 1855) had significantly higher nAb response rates and GM ID50 titers (response odds ratio (OR) = 14.14, 95% CI: 1.90, 1789.08, p = 0.004, q = 0.036; GMR = 2.91, 95% CI: 1.65, 5.14, p < 0.001, q = 0.001). Participants with high BMI (≥30 vs < 30; p = 0.002, q = 0.005) and participants living with diabetes (Yes vs No; p = 0.019, q = 0.031) had more than double the GM ID50 titers of their lower BMI or diabetes-free counterparts (BMI: GMR = 2.12, 95% CI: 1.32, 3.40; diabetes: GMR = 2.17, 95% CI: 1.14, 4.16) (Table 2). Sex assigned at birth, race, and some medical comorbidities (hypertension; and any of COPD, emphysema, or asthma) were not associated with ID50 titers.

Results were generally similar and conclusions unchanged based on an analysis of ID80 nAb titers (S2 Table).

### Neutralizing antibody kinetics relative to COVID-19 waves

**Fig 2:**
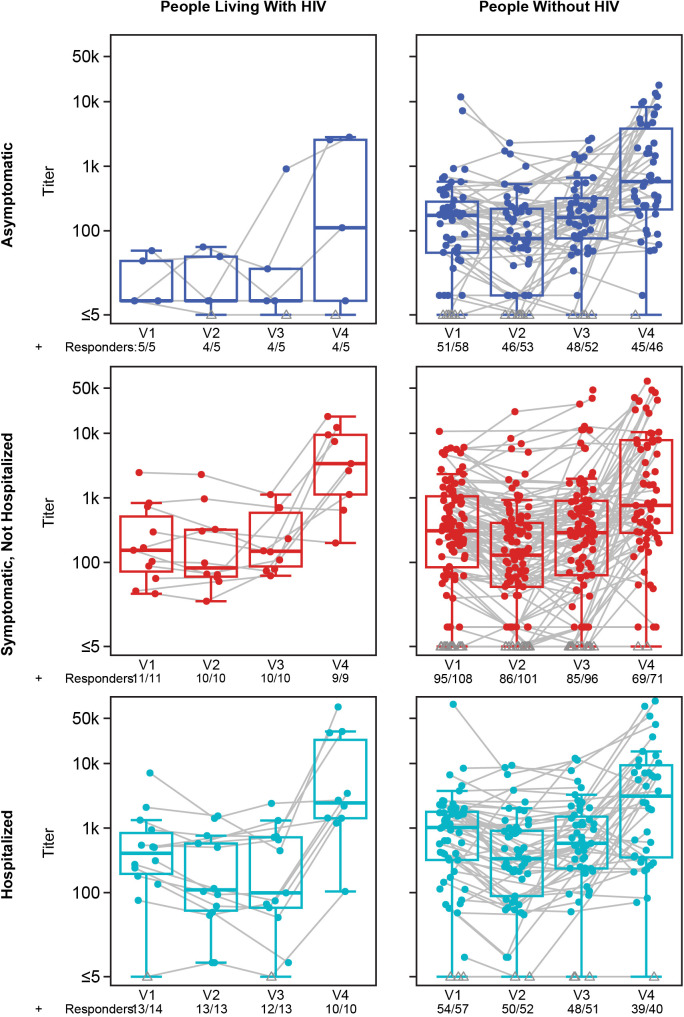
Distribution and traces of individual SARS-CoV-2 neutralizing antibody (nAb) responses over time by participant HIV status and COVID-19 severity. nAb ID50 titers were measured at enrollment (V1) and approximately 2 months (V2), 4 months (V3), and 12 months (V4) thereafter. Boxplots indicate the median (middle bar), interquartile range (box length), the most extreme data points that are no more than 1.5 times the interquartile range, or if no value meets this criterion, to the data extremes (whiskers).

Neutralizing antibody kinetics were evaluated with measurement of titers from samples collected at Visit 1 (enrollment) and Visits 2, 3, and 4, corresponding to approximately 2, 3, and 12 months after enrollment (Fig 2; S2 Table). Among both PLWH and PWOH and in all COVID-19 severity groups, nAb titers tended to decline from Visit 1 to Visit 2. There tended to be an increase in neutralization titers between Visits 2 and 3, and an even higher increase from Visit 3 to Visit 4.

**Fig 3:**
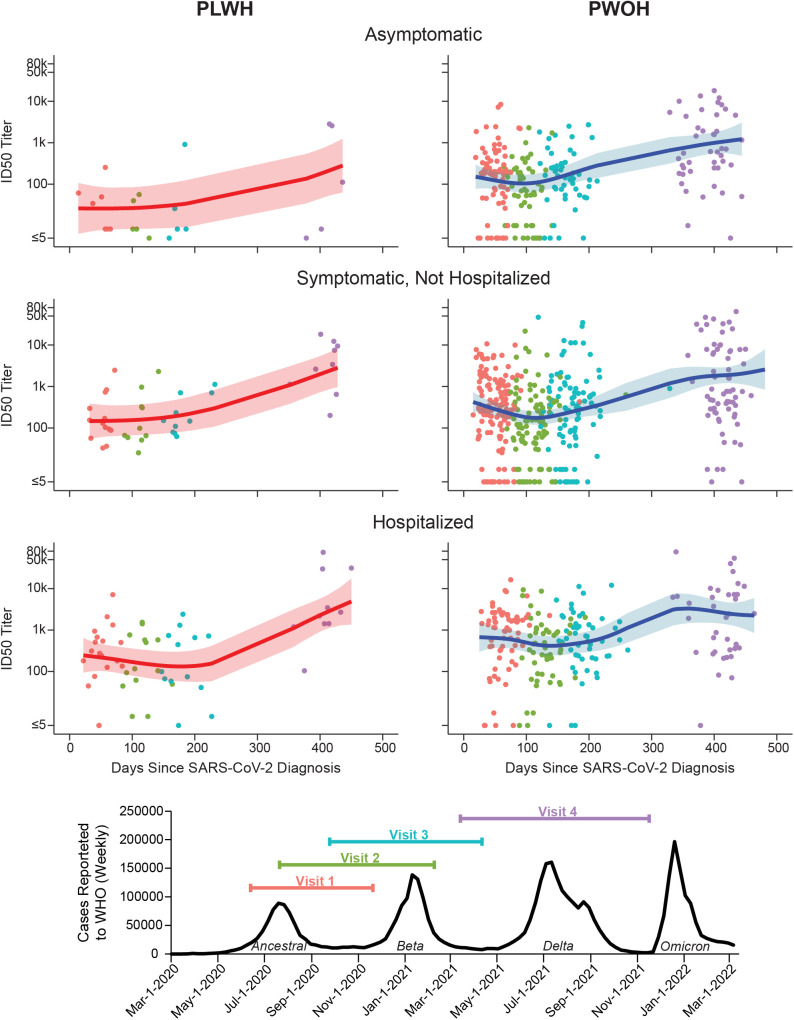
Average SARS-CoV-2 neutralizing antibody trajectory by HIV status and COVID-19 severity group. Individual nAb ID50 titers are shown as well as population geometric mean trajectories and 95% confidence bands estimated using a generalized additive mixed model (GAMM). Bottom panel shows the total number of COVID-19 cases reported to the WHO in the countries included in this analysis over calendar time, with the study visit intervals overlaid. Symptomatic participants and participants hospitalized due to COVID-19 were eligible to enroll 1-8 weeks after COVID-19 disease resolution. Asymptomatic participants were eligible to enroll 2-10 weeks after SARS-CoV-2 acquisition diagnosis. Visit 1 was the enrollment visit. Visits 2, 3, and 4 occurred at 2, 4, and 12 months after enrollment, respectively.

Population-average trajectories in SARS-CoV-2 nAb responses were considered in the context of waves of SARS-CoV-2 acquisition in the communities where the study was conducted (Fig 3). nAb ID50 titers generally decreased between Visit 1 and the subsequent 100 days, after which there was a steady increase. The increase in nAb responses observed between Visits 3 and 4, at approximately 12 months after enrollment, coincided with the large Delta COVID-19 wave in southern Africa in the second half of 2021 (Fig 3). In addition, starting in February 2021, southern African countries started their national COVID-19 vaccination programs, coinciding with Visit 3 and Visit 4 windows.

### Neutralizing antibodies in participants with and without HIV

While positive nAb response rates were similar between PLWH and PWOH (Odds Ratio [OR] = 1.97 [95% CI: 0.56, 10.44], p = 0.317, q = 0.718; Table 2), the 39 PLWH had an estimated 65% lower geometric mean anti-SARS-CoV-2 nAb ID50 titer at enrollment compared to PWOH (GMR = 0.35 [95% CI: 0.18, 0.69], p = 0.003, q = 0.006), after adjusting for age, sex assigned at birth, region, days since SARS-CoV-2 diagnosis, and COVID-19 severity. This difference in the GM ID50 titer by HIV status was apparent among asymptomatic (PLWH = 28.3 [95%CI: 8.4, 95.1] vs. PWOH = 141.1 [95%CI: 91.0, 219.9]), symptomatic (PLWH = 168.3 [95%CI: 77.8, 363.9] vs. PWOH = 313.1 [95%CI:217.5, 450.7]), and hospitalized (PLWH = 240.3 [95%CI: 100.5, 574.9] vs. PWOH = 689.1 [95%CI: 432.8, 1097.3]) participants (S3 Table).

These trends between PLWH and PWOH generally persisted over time: PLWH and PWOH continued to exhibit similar response rates, and PLWH had lower or comparable titers across Visits 2, 3, and 4. At Visit 2, approximately 2 months after enrollment, the GM ID50 titer was 15.0 (95% CI: 4.0, 57.0) among asymptomatic PLWH vs. 70.4 (95% CI: 44.2, 112.2) among PWOH, 153.0 (95% CI: 54.4, 430.2) vs. 129.6 (95% CI: 82.9, 202.8) among symptomatic PLWH vs. PWOH, and 150.1 (95% CI: 55.3, 407.6) vs. 322.0 (95% CI: 200.1, 518.2) among hospitalized PLWH vs. PWOH (S3 Table). At Visit 3, approximately 3–4 months after enrollment, GM ID50 titers among PLWH vs. PWOH were 24.0 (95% CI: 1.7, 333.1) vs. 151.8 (95% CI: 98.7, 233.7) among asymptomatic; 202.3 (95% CI: 95.4, 428.9) vs. 252.4 (95% CI: 156.6, 406.7) among symptomatic, non-hospitalized; and 152.5 (95% CI: 47.6, 489.3) vs. 529.7 (95% CI: 325.4, 862.3) among hospitalized individuals. Thus, while both groups experienced elevations in their ancestral strain antibodies during follow-up, titers in PLWH generally stayed lower.

Results were similar and conclusions unchanged based on an analysis of ID80 titers (S4 Table).

## Discussion

This work characterized the SARS-CoV-2-specific nAb response of a diverse population across four countries in southern Africa (South Africa, Zimbabwe, Zambia, and Malawi) shortly after SARS-CoV-2 diagnosis during the early stages of the pandemic (between June 2020 and January 2021). Importantly, this study evaluated nAb responses elicited after primary SARS-CoV-2 acquisition, stratified according to COVID-19 severity and emphasizing comparisons between participants living with and without HIV.

NAb response rates at enrollment were consistently high across baseline characteristics, ranging from 80% to a high of 100% among the 58 individuals over age 55. Consistent with convalescence from acquisition approximately 6 weeks prior, geometric mean nAb titers at enrollment were more variable between groups, with age > 55 years old, disease severity, diabetes, high BMI, and not smoking associated with higher magnitude responses. Consistent with the results from the Americas cohort [34], most participants exhibited a steady decrease in nAb titers between enrollment and the next visit, approximately 100 days thereafter, although this trend was less pronounced in the cohort of PLWH. Beyond that and through 12 months (Visit 4), nAb titers generally increased over time, which suggests new antigenic stimulation given that antibodies would be expected to exhibit continual decline [34,44,45]. This phenomenon could be the result of a number of external factors, most notably the continuing waves of acquisition and the beginning of vaccine rollout. There were appreciable peaks in SARS-CoV-2 acquisition rates based on population-level epidemiological data [1] for the contributing countries; these peaks corresponded to the Beta SARS-CoV-2 variant during the timeframe of Visits 2 and 3 (around 2 and 4 months, respectively), and to the Delta variant during the timeframe of Visit 4 (around 12 months). Clinical resources (e.g., test kits) were still strained during these times, so confirmatory testing was rare.

Many of our findings are consistent with those published from the Americas cohort of this same trial [34], and with the broader literature, though some differences were observed. In particular, the associations we found between nAb responses to SARS-CoV-2 acquisition and disease severity, diabetes, BMI, and age have also been documented elsewhere [15,34,46]. In contrast, the lack of an association with sex assigned at birth and hypertension contrasts with several published reports [34,47–49]. The consistency between our results and prior literature is of particular importance in the context of an overall dearth of SARS-CoV-2 immunogenicity data published from trials conducted in southern Africa. It is noteworthy that the factors above remain associated with nAb responses irrespective of geography and a myriad of other societal, cultural, and potentially clinical factors.

The cohort of PLWH acutely infected with SARS-CoV-2 in southern Africa was a unique strength of this study. While SARS-CoV-2-specific nAb response rates were similar between PLWH and PWOH, PLWH had nAb ID50 titers that were, on average, approximately one-third lower than those of PWOH at enrollment, consistent with data from the Americas cohort of this study showing reduced immunogenicity in PLWH [25]. Interestingly, this effect was not solely driven by any single severity category, as both extremes (asymptomatic and hospitalized) showed significant differences between PLWH and PWOH. Across follow-up visits there was more variability, and it was not possible to disentangle potential differences between PLWH and PWOH resulting from waning immunity, possible reacquisition, or early vaccination. The variability at later time points may have been influenced by potential differences in COVID-19 vulnerability and vaccine uptake in PLWH. Nevertheless, PLWH who were asymptomatic tended to have lower nAb titers than asymptomatic PWOH across all four study visits.

The results from this study must be viewed in the context of various limitations. Some of these limitations were a consequence of the timing of the trial: the study protocol was written in the earliest stages of the pandemic and conducted when there was still pervasive uncertainty surrounding SARS-CoV-2 acquisition and pathogenesis. Healthcare resources and infrastructure were stressed, resulting in considerable constraints and inconsistencies in practices at clinical facilities across the globe. Specific to this cohort, the threshold for hospitalization was likely inconsistent, as hospitals needed to prioritize the most severe cases and admittance would therefore have been subject to local case fluctuations and capacity. Nonessential person-to-person contact was minimized as a part of pandemic-related protections instituted by the study or clinical research institutional policies, and as a result some study procedures were conducted by phone and in-person portions were often substantially time-limited, which limited more comprehensive data collection. Data regarding post-enrollment SARS-CoV-2 reacquisition was rarely collected due to limited accessibility of SARS-CoV-2 testing kits and policies limiting the type of allowable research clinic procedures early in the pandemic, and vaccine access and reporting was inconsistent during the study period. These limitations impaired our ability to precisely characterize the impacts of reacquisition and vaccination on neutralizing antibody trajectories, particularly through the last two study visits that took place during or after the Beta and Delta waves and the gradual introduction of vaccines. Similarly, relevant HIV data, including viral loads, CD4 counts, and antiretroviral status were rarely available due to pandemic-related stress on healthcare infrastructure and concomitant disruptions in the conduct of otherwise routine follow-up care, including viral load monitoring and sharing of medical records. These HIV-related data would be useful in more accurately characterizing the PLWH cohort. Though we do identify distinct elements of the immunologic profile in PLWH compared to PWOH, this remains an incompletely resolved area of interest, as some studies have reported similar SARS-CoV-2-specific antibody responses in PLWH and PWOH [23,25,50–52], while others have reported reduced responses in PLWH [25,53]. Furthermore, as an observational cohort study, the differences we observed in nAb responses between groups may be subject to bias due to confounding; while analyses adjusted for factors measured and anticipated to affect nAb responses, there may be others that were not controlled for or measured. There was also a survivorship bias in the study population, as participants were only eligible to enroll after disease resolution or at least two weeks after confirmed asymptomatic SARS-CoV-2 acquisition.

Altogether, these results provide an important characterization of neutralizing antibody responses to primary SARS-CoV-2 acquisition—a unique endpoint as SARS-CoV-2-naïve individuals are an increasing rarity, globally—immediately following recovery and over time in southern Africa early during the COVID-19 pandemic. Factors explaining variability in the antibody responses align well with those identified in other populations and regions of the world. Moreover, the results provide additional evidence that people living with HIV in Africa do mount a more limited neutralizing antibody response to SARS-CoV-2 acquisition, suggesting timely boosters and additional precautions to limit exposure may be prudent.

## Supporting information

S1 Text Study inclusion and exclusion criteria. (DOCX)

S2 Text Vesicular stomatitis virus (VSV) antibody assay details. (DOCX)

S3 Text Calibration of neutralizing antibody assays. (DOCX)

S1 Fig Scatterplot of neutralizing antibody (nAb) responses from the VSV assay before and after calibration vs. neutralizing antibody (nAb) responses for the 293T/ACE2 assay, based on the calibration cohort. (DOCX)

S1 Table Estimated anti-SARS-CoV-2 neutralizing antibody (nAb) response rate and geometric mean (GM) ID50/ID80 titer at enrollment by selected baseline participant characteristics. (DOCX)

S2 Table Results of multivariate modeling associating baseline participant characteristics with SARS-CoV-2 neutralizing antibody (nAb) ID80 titer at enrollment. Each predictor of interest is studied independently for its association with the nAb ID80 titer (using log-linear regression). Each regression model adjusts for confounders: COVID-19 severity, age, sex at birth, African region, and days since SARS-CoV-2 diagnosis.
(DOCX)

S3 Table Estimated anti-SARS-CoV-2 neutralizing antibody (nAb) response rate and geometric mean (GM) ID50 titer by visit among people living with HIV (PLWH) and people without HIV (PWOH) by COVID-19 severity group. (DOCX)

S4 Table Estimated anti-SARS-CoV-2 neutralizing antibody (nAb) response rate and geometric mean (GM) ID80 titer by visit among people living with HIV (PLWH) and people without HIV (PWOH) by COVID-19 severity group. (DOCX)

## References

[1] World Health Organization. WHO Coronavirus (COVID-19) dashboard [cited 2024 September 18]. Available from: https://data.who.int/dashboards/covid19/cases

[2] Gilbert PB, Donis RO, Koup RA, Fong Y, Plotkin SA, Follmann D. A Covid-19 Milestone Attained - A Correlate of Protection for Vaccines. N Engl J Med. 2022;387(24):2203–6. doi: 10.1056/NEJMp2211314 36507702

[3] Khoury DS, Schlub TE, Cromer D, Steain M, Fong Y, Gilbert PB, et al. Correlates of Protection, Thresholds of Protection, and Immunobridging among Persons with SARS-CoV-2 Infection. Emerg Infect Dis. 2023;29(2):381–8. doi: 10.3201/eid2902.221422 36692375 PMC9881762

[4] Atti A, Insalata F, Carr EJ, Otter AD, Castillo-Olivares J, Wu M, et al. Antibody correlates of protection from SARS-CoV-2 reinfection prior to vaccination: A nested case-control within the SIREN study. J Infect. 2022;85(5):545–56. doi: 10.1016/j.jinf.2022.09.004 36089104 PMC9458758

[5] Castilla J, Lecea Ó, Martín Salas C, Quílez D, Miqueleiz A, Trobajo-Sanmartín C, et al. Seroprevalence of antibodies against SARS-CoV-2 and risk of COVID-19 in Navarre, Spain, May to July 2022. Euro Surveill. 2022;27(33):2200619. doi: 10.2807/1560-7917.ES.2022.27.33.2200619 35983774 PMC9389855

[6] Gilboa M, Gonen T, Barda N, Cohn S, Indenbaum V, Weiss-Ottolenghi Y, et al. Factors Associated With Protection From SARS-CoV-2 Omicron Variant Infection and Disease Among Vaccinated Health Care Workers in Israel. JAMA Netw Open. 2023;6(5):e2314757. doi: 10.1001/jamanetworkopen.2023.14757 37219906 PMC10208153

[7] Holmer HK, Mackey K, Fiordalisi CV, Helfand M. Major Update 2: Antibody Response and Risk for Reinfection After SARS-CoV-2 Infection-Final Update of a Living, Rapid Review. Ann Intern Med. 2023;176(1):85–91. doi: 10.7326/M22-1745 36442059 PMC9707440

[8] Yamamoto S, Oshiro Y, Inamura N, Nemoto T, Tan T, Horii K, et al. Correlates of Nucleocapsid Antibodies and a Combination of Spike and Nucleocapsid Antibodies Against Protection of SARS-CoV-2 Infection During the Omicron XBB.1.16/EG.5-Predominant Wave. Open Forum Infect Dis. 2024;11(9):ofae455. doi: 10.1093/ofid/ofae455 39220657 PMC11363870

[9] Zhang B, Fong Y, Fintzi J, Chu E, Janes HE, Kenny A, et al. Omicron COVID-19 immune correlates analysis of a third dose of mRNA-1273 in the COVE trial. Nat Commun. 2024;15(1):7954. doi: 10.1038/s41467-024-52348-9 39261482 PMC11390939

[10] Sun K, Bhiman JN, Tempia S, Kleynhans J, Madzorera VS, Mkhize Q, et al. SARS-CoV-2 correlates of protection from infection against variants of concern. Nat Med. 2024;30(10):2805–12. doi: 10.1038/s41591-024-03131-2 39060660 PMC11533127

[11] Lasrado N, Barouch DH. SARS-CoV-2 Hybrid Immunity: The Best of Both Worlds. J Infect Dis. 2023;228(10):1311–3. doi: 10.1093/infdis/jiad353 37592872

[12] Bobrovitz N, Ware H, Ma X, Li Z, Hosseini R, Cao C, et al. Protective effectiveness of previous SARS-CoV-2 infection and hybrid immunity against the omicron variant and severe disease: a systematic review and meta-regression. Lancet Infect Dis. 2023;23(5):556–67. doi: 10.1016/S1473-3099(22)00801-5 36681084 PMC10014083

[13] Altarawneh HN, Chemaitelly H, Ayoub HH, Tang P, Hasan MR, Yassine HM, et al. Effects of previous infection, vaccination, and hybrid immunity against symptomatic Alpha, Beta, and Delta SARS-CoV-2 infections: an observational study. EBioMedicine. 2023;95:104734. doi: 10.1016/j.ebiom.2023.104734 37515986 PMC10404859

[14] Garrett N, Tapley A, Hudson A, Dadabhai S, Zhang B, Mgodi NM, et al. Hybrid versus vaccine immunity of mRNA-1273 among people living with HIV in East and Southern Africa: a prospective cohort analysis from the multicentre CoVPN 3008 (Ubuntu) study. EClinicalMedicine. 2025;80:103054. doi: 10.1016/j.eclinm.2024.103054 39902315 PMC11788791

[15] Vanshylla K, Di Cristanziano V, Kleipass F, Dewald F, Schommers P, Gieselmann L, et al. Kinetics and correlates of the neutralizing antibody response to SARS-CoV-2 infection in humans. Cell Host Microbe. 2021;29(6):917-929.e4. doi: 10.1016/j.chom.2021.04.015 33984285 PMC8090990

[16] Legros V, Denolly S, Vogrig M, Boson B, Siret E, Rigaill J, et al. A longitudinal study of SARS-CoV-2-infected patients reveals a high correlation between neutralizing antibodies and COVID-19 severity. Cell Mol Immunol. 2021;18(2):318–27. doi: 10.1038/s41423-020-00588-2 33408342 PMC7786875

[17] Bertagnolio S, Thwin SS, Silva R, Nagarajan S, Jassat W, Fowler R, et al. Clinical features of, and risk factors for, severe or fatal COVID-19 among people living with HIV admitted to hospital: analysis of data from the WHO Global Clinical Platform of COVID-19. Lancet HIV. 2022;9(7):e486–95. doi: 10.1016/S2352-3018(22)00097-2 35561704 PMC9090268

[18] Mellor MM, Bast AC, Jones NR, Roberts NW, Ordóñez-Mena JM, Reith AJM, et al. Risk of adverse coronavirus disease 2019 outcomes for people living with HIV. AIDS. 2021;35(4):F1–10. doi: 10.1097/QAD.0000000000002836 33587448 PMC7924978

[19] Danwang C, Noubiap JJ, Robert A, Yombi JC. Outcomes of patients with HIV and COVID-19 co-infection: a systematic review and meta-analysis. AIDS Res Ther. 2022;19(1):3. doi: 10.1186/s12981-021-00427-y 35031068 PMC8759058

[20] Ssentongo P, Heilbrunn ES, Ssentongo AE, Advani S, Chinchilli VM, Nunez JJ, et al. Epidemiology and outcomes of COVID-19 in HIV-infected individuals: a systematic review and meta-analysis. Sci Rep. 2021;11(1):6283. doi: 10.1038/s41598-021-85359-3 33737527 PMC7973415

[21] Dong Y, Li Z, Ding S, Liu S, Tang Z, Jia L, et al. HIV infection and risk of COVID-19 mortality: A meta-analysis. Medicine (Baltimore). 2021;100(26):e26573. doi: 10.1097/MD.0000000000026573 34190201 PMC8257842

[22] Mondi A, Cimini E, Colavita F, Cicalini S, Pinnetti C, Matusali G, et al. COVID-19 in people living with HIV: Clinical implications of dynamics of the immune response to SARS-CoV-2. J Med Virol. 2021;93(3):1796–804. doi: 10.1002/jmv.26556 32975842 PMC7537181

[23] Spinelli MA, Lynch KL, Yun C, Glidden DV, Peluso MJ, Henrich TJ, et al. SARS-CoV-2 seroprevalence, and IgG concentration and pseudovirus neutralising antibody titres after infection, compared by HIV status: a matched case-control observational study. Lancet HIV. 2021;8(6):e334–41. doi: 10.1016/S2352-3018(21)00072-2 33933189 PMC8084354

[24] Ambrosioni J, Blanco JL, Reyes-Urueña JM, Davies M-A, Sued O, Marcos MA, et al. Overview of SARS-CoV-2 infection in adults living with HIV. Lancet HIV. 2021;8(5):e294–305. doi: 10.1016/S2352-3018(21)00070-9 33915101 PMC8075775

[25] Schuster DJ, Karuna S, Brackett C, Wesley M, Li SS, Eisel N, et al. Lower SARS-CoV-2-specific humoral immunity in people living with HIV-1 recovered from nonhospitalized COVID-19. JCI Insight. 2022;7(21):e158402. doi: 10.1172/jci.insight.158402 36136590 PMC9675463

[26] Motsoeneng BM, Bhiman JN, Richardson SI, Moore PL. SARS-CoV-2 humoral immunity in people living with HIV-1. Trends Immunol. 2024;45(7):511–22. doi: 10.1016/j.it.2024.05.005 38890026

[27] UNAIDS. UNAIDS Global Factsheets 2023 [cited 2024 August 8]. Available from: https://aidsinfo.unaids.org/

[28] Maeda JM, Nkengasong JN. The puzzle of the COVID-19 pandemic in Africa. Science. 2021;371(6524):27–8. doi: 10.1126/science.abf8832 33384364

[29] Bwire G, Ario AR, Eyu P, Ocom F, Wamala JF, Kusi KA, et al. The COVID-19 pandemic in the African continent. BMC Med. 2022;20(1):167. doi: 10.1186/s12916-022-02367-4 35501853 PMC9059455

[30] Salyer SJ, Maeda J, Sembuche S, Kebede Y, Tshangela A, Moussif M, et al. The first and second waves of the COVID-19 pandemic in Africa: a cross-sectional study. Lancet. 2021;397(10281):1265–75. doi: 10.1016/S0140-6736(21)00632-2 33773118 PMC8046510

[31] Obande GA, Bagudo AI, Mohamad S, Deris ZZ, Harun A, Yean CY, et al. Current State of COVID-19 Pandemic in Africa: Lessons for Today and the Future. Int J Environ Res Public Health. 2021;18(19):9968. doi: 10.3390/ijerph18199968 34639270 PMC8507711

[32] Massinga Loembé M, Tshangela A, Salyer SJ, Varma JK, Ouma AEO, Nkengasong JN. COVID-19 in Africa: the spread and response. Nat Med. 2020;26(7):999–1003. doi: 10.1038/s41591-020-0961-x 32528154

[33] Soy A. Coronavirus in Africa: Five reasons why Covid-19 has been less deadly than elsewhere bbc.com 2020 [cited 2024 September 16]. Available from: https://www.bbc.com/news/world-africa-54418613

[34] Karuna S, Li SS, Grant S, Walsh SR, Frank I, Casapia M, et al. Neutralizing antibody responses over time in demographically and clinically diverse individuals recovered from SARS-CoV-2 infection in the United States and Peru: A cohort study. PLoS Med. 2021;18(12):e1003868. doi: 10.1371/journal.pmed.1003868 34871308 PMC8687542

[35] Karuna S, Gallardo-Cartagena JA, Theodore D, Hunidzarira P, Montenegro-Idrogo J, Hu J, et al. Post-COVID symptom profiles and duration in a global convalescent COVID-19 observational cohort: Correlations with demographics, medical history, acute COVID-19 severity and global region. J Glob Health. 2023;13:06020. doi: 10.7189/jogh.13.06020 37352144 PMC10289480

[36] Shen X, Tang H, McDanal C, Wagh K, Fischer W, Theiler J, et al. SARS-CoV-2 variant B.1.1.7 is susceptible to neutralizing antibodies elicited by ancestral spike vaccines. Cell Host Microbe. 2021;29(4):529-539.e3. doi: 10.1016/j.chom.2021.03.002 33705729 PMC7934674

[37] Sholukh AM, Fiore-Gartland A, Ford ES, Miner MD, Hou YJ, Tse LV, et al. Evaluation of Cell-Based and Surrogate SARS-CoV-2 Neutralization Assays. J Clin Microbiol. 2021;59(10):e0052721. doi: 10.1128/JCM.00527-21 34288726 PMC8451402

[38] Whitt MA. Generation of VSV pseudotypes using recombinant ΔG-VSV for studies on virus entry, identification of entry inhibitors, and immune responses to vaccines. J Virol Methods. 2010;169(2):365–74. doi: 10.1016/j.jviromet.2010.08.006 20709108 PMC2956192

[39] Zhao X, Howell KA, He S, Brannan JM, Wec AZ, Davidson E, et al. Immunization-Elicited Broadly Protective Antibody Reveals Ebolavirus Fusion Loop as a Site of Vulnerability. Cell. 2017;169(5):891-904.e15. doi: 10.1016/j.cell.2017.04.038 28525756 PMC5803079

[40] Heinze G, Schemper M. A solution to the problem of separation in logistic regression. Stat Med. 2002;21(16):2409–19. doi: 10.1002/sim.1047 12210625

[41] Benjamini Y, Hochberg Y. Controlling the False Discovery Rate: A Practical and Powerful Approach to Multiple Testing. Journal of the Royal Statistical Society Series B: Statistical Methodology. 1995;57(1):289–300. doi: 10.1111/j.2517-6161.1995.tb02031.x

[42] Wood SN. Low-rank scale-invariant tensor product smooths for generalized additive mixed models. Biometrics. 2006;62(4):1025–36. doi: 10.1111/j.1541-0420.2006.00574.x 17156276

[43] R Core Team. R: A Language and Environment for Statistical Computing. Vienna, Austria: R Foundation for Statistical Computing. 2021.

[44] Seow J, Graham C, Merrick B, Acors S, Pickering S, Steel KJA, et al. Longitudinal observation and decline of neutralizing antibody responses in the three months following SARS-CoV-2 infection in humans. Nat Microbiol. 2020;5(12):1598–607. doi: 10.1038/s41564-020-00813-8 33106674 PMC7610833

[45] Huang Y-F, Hsu F-C, Wu J-J, Lin Y-L, Liu M-T, Yang C-H, et al. Longitudinal neutralizing antibody responses after SARS-CoV-2 infection: A convalescent cohort study in Taiwan. J Microbiol Immunol Infect. 2023;56(3):506–15. doi: 10.1016/j.jmii.2023.03.004 36967265 PMC10019033

[46] Chen X, Pan Z, Yue S, Yu F, Zhang J, Yang Y, et al. Disease severity dictates SARS-CoV-2-specific neutralizing antibody responses in COVID-19. Signal Transduct Target Ther. 2020;5(1):180. doi: 10.1038/s41392-020-00301-9 32879307 PMC7464057

[47] Markmann AJ, Giallourou N, Bhowmik DR, Hou YJ, Lerner A, Martinez DR, et al. Sex Disparities and Neutralizing-Antibody Durability to SARS-CoV-2 Infection in Convalescent Individuals. mSphere. 2021;6(4):e0027521. doi: 10.1128/mSphere.00275-21 34431693 PMC8386415

[48] Klein SL, Pekosz A, Park H-S, Ursin RL, Shapiro JR, Benner SE, et al. Sex, age, and hospitalization drive antibody responses in a COVID-19 convalescent plasma donor population. J Clin Invest. 2020;130(11):6141–50. doi: 10.1172/JCI142004 32764200 PMC7598041

[49] Wu F, Liu M, Wang A, Lu L, Wang Q, Gu C, et al. Evaluating the Association of Clinical Characteristics With Neutralizing Antibody Levels in Patients Who Have Recovered From Mild COVID-19 in Shanghai, China. JAMA Intern Med. 2020;180(10):1356–62. doi: 10.1001/jamainternmed.2020.4616 32808970 PMC9377417

[50] Khan K, Lustig G, Bernstein M, Archary D, Cele S, Karim F, et al. Immunogenicity of Severe Acute Respiratory Syndrome Coronavirus 2 (SARS-CoV-2) Infection and Ad26.CoV2.S Vaccination in People Living With Human Immunodeficiency Virus (HIV). Clin Infect Dis. 2022;75(1):e857–64. doi: 10.1093/cid/ciab1008 34893824 PMC8689810

[51] Hwa S-H, Snyman J, Bernstein M, Ganga Y, Cele S, Muema D, et al. Association Between Human Immunodeficiency Virus Viremia and Compromised Neutralization of Severe Acute Respiratory Syndrome Coronavirus 2 Beta Variant. J Infect Dis. 2023;227(2):211–20. doi: 10.1093/infdis/jiac343 35975942 PMC9452105

[52] Höft MA, Burgers WA, Riou C. The immune response to SARS-CoV-2 in people with HIV. Cell Mol Immunol. 2024;21(2):184–96. doi: 10.1038/s41423-023-01087-w 37821620 PMC10806256

[53] Alrubayyi A, Gea-Mallorquí E, Touizer E, Hameiri-Bowen D, Kopycinski J, Charlton B, et al. Characterization of humoral and SARS-CoV-2 specific T cell responses in people living with HIV. Nat Commun. 2021;12(1):5839. doi: 10.1038/s41467-021-26137-7 34611163 PMC8492866

