# Supplementary material for: Neutralizing antibody responses over time in a demographically and clinically diverse cohort of individuals recovered from SARS-CoV-2 acquisition in Africa: A cohort study
Source: PLOS Glob Public Health. 2025 Sep 11;5(9):e0005156. doi: 10.1371/journal.pgph.0005156 (PMC12425307; doi:10.1371/journal.pgph.0005156)
Supplement: S1 Text — (DOCX) [file pgph.0005156.s001.docx]

**S1 Text. Study inclusion and exclusion criteria**

Inclusion criteria

1. Age 18 or older.

2. Reports having had a positive test for SARS-CoV-2.

3. Reports resolution of COVID-19 within 1-8 weeks of enrollment OR, if asymptomatic infection, reports positive SARS-CoV-2 test within 2-10 weeks of enrollment. Not excluded: individuals with symptoms consistent with residual sequelae of resolved COVID-19, in the clinical judgement of the investigator.

4. Access to a participating HVTN or HPTN Clinical Research Site and willingness to be followed for the planned duration of the study.

5. Ability and willingness to provide informed consent.

6. Assessment of understanding: volunteer demonstrates understanding of this study.

7. Volunteers who were assigned female sex at birth: negative urine or serum beta human chorionic gonadotropin (β-HCG) pregnancy test within 4 days of enrollment visit (i.e., prior to enrollment blood draw or nasal collections). Persons who are NOT of reproductive potential due to having undergone hysterectomy or bilateral oophorectomy (verified by medical records) or having reached menopause (no menses for ≥ 1 year), are not required to undergo pregnancy testing.

Exclusion criteria

1. Reports current COVID-19.

2. Pregnant.

3. Receipt of SARS-CoV-2 specific antibodies (eg, convalescent plasma or sera, monoclonal antibodies, hyperimmune globulin). Not excluded: antibody therapy without SARS-CoV-2 specificity (eg, IL-6 pathway inhibitors for COVID-19).

4. SARS-CoV-2 vaccine(s) received in a prior vaccine trial.

5. Any medical, psychiatric, occupational, or other condition that, in the judgment of the investigator, would interfere with, or serve as a contraindication to, protocol adherence or a volunteer’s ability to give informed consent.
