# Supplementary material for: Neutralizing antibody responses over time in a demographically and clinically diverse cohort of individuals recovered from SARS-CoV-2 acquisition in Africa: A cohort study
Source: PLOS Glob Public Health. 2025 Sep 11;5(9):e0005156. doi: 10.1371/journal.pgph.0005156 (PMC12425307; doi:10.1371/journal.pgph.0005156)
Supplement: S2 Text — (DOCX) [file pgph.0005156.s002.docx]

**S2 Text. Vesicular stomatitis virus (VSV) antibody assay details**

The VSV assay was used for samples from PLWH, as antiretroviral therapy interferes with the 293T/ACE2 assay. Vero cells were seeded at 2x10^4^ cells/well in black­walled 96­well plates 24 hours before the assay was performed. VSV pseudovirus was prepared using a codon­optimized gene of SARS­CoV-2 Spike protein (YP_009724390.1) cloned into pcDNA3.1 (PsVSV­Luc­D19) and VSV(*G**∆G-luciferase) system purchased from Kerafast (Boston, MA) [1, 2]. VSV(*G**∆G­luciferase) pseudotyped with SARS-CoV-2 Spike was produced in 293T cells and stored at -80°C. Luciferase activity was measured by Bio­Glo Luciferase Assay System (Promega, Madison, WI) using a 2030 VICTOR X3 multilabel reader (PerkinElmer, Waltham, MA). Median tissue culture infectious dose (TCID_50_) was measured using serial 2-fold dilutions of the prepared pseudovirus. Percent neutralization was calculated by the following equation: [1– (RLU with virus+sample – RLU with uninfected cells) / (RLU with virus only cells – RLU with uninfected cells] x 100. Plasma collected from an individual outside the study with severe, PCR­-confirmed SARS­-CoV­-2 infection collected after hospital release was used as a positive control. Pooled human serum collected in 2015­2018 (prior to the COVID-19 pandemic) was used as a negative control. Notably, the SARS-CoV-2 Spike used in the VSV pseudovirus assay was the original Wuhan strain D614, not SARS-CoV-2 D614G. Nonetheless, no discernible difference in infectivity and neutralization between Wuhan strain D614 and SARS­-CoV­-2 D614G has been identified [3].

The response was measured by the 50% (or 80%) inhibitory titer (ID50, ID80), defined as the serum dilution that corresponded to 50% (80%) neutralization. If neutralization never reached 50% (80%), ID50 (ID80) was estimated in GraphPad Prism v9.0 using 4­-parameter logistic regression (4PL). If the estimation failed, the ID50 (ID80) was set to 10.

**References**

1. Whitt MA. Generation of VSV pseudotypes using recombinant DeltaG-VSV for studies on virus entry, identification of entry inhibitors, and immune responses to vaccines. J Virol Methods. 2010;169(2):365-74. Epub 20100813. doi: 10.1016/j.jviromet.2010.08.006. PubMed PMID: 20709108; PubMed Central PMCID: PMCPMC2956192.

2. Zhao X, Howell KA, He S, Brannan JM, Wec AZ, Davidson E, et al. Immunization-Elicited Broadly Protective Antibody Reveals Ebolavirus Fusion Loop as a Site of Vulnerability. Cell. 2017;169(5):891-904 e15. doi: 10.1016/j.cell.2017.04.038. PubMed PMID: 28525756; PubMed Central PMCID: PMCPMC5803079.

3. Sholukh AM, Fiore-Gartland A, Ford ES, Miner MD, Hou YJ, Tse LV, et al. Evaluation of Cell-Based and Surrogate SARS-CoV-2 Neutralization Assays. J Clin Microbiol. 2021;59(10):e0052721. Epub 20210721. doi: 10.1128/JCM.00527-21. PubMed PMID: 34288726; PubMed Central PMCID: PMCPMC8451402.
