# Supplementary material for: Neutralizing antibody responses over time in a demographically and clinically diverse cohort of individuals recovered from SARS-CoV-2 acquisition in Africa: A cohort study
Source: PLOS Glob Public Health. 2025 Sep 11;5(9):e0005156. doi: 10.1371/journal.pgph.0005156 (PMC12425307; doi:10.1371/journal.pgph.0005156)
Supplement: S3 Text — (DOCX) [file pgph.0005156.s003.docx]

**S3 Text. Calibration of neutralizing antibody assays**

In order to combine the nAb data measured using the 293T/ACE2 assay for participants without HIV with the data measured using the VSV assay for PLWH, we used nAb data measured by both assays for a separate “calibration cohort”. The calibration cohort consisted of 216 individuals without HIV from the HVTN 405 Americas cohort. Approach 3 of Huang et al., 2021 was adopted [1]. Specifically, a linear regression model was used to regress the log transformed readouts from the 293T/ACE2 assay (y) on the log transformed readouts from the VSV assay (x), i.e. *log*(*y*) *α*+*β ∗ log*(*x*) among the samples with titers above the lower limit of detection for both assays. Estimates of the calibration parameters *α* and *β* were then used to calibrate the responses from PLWH, measured using the VSV assay, to the scale of the responses for participants without HIV, measured using the 293T/ACE2 assay. For responses that were negative on the VSV assay, the response was also considered to be negative on the 293T/ACE2 assay and the response was set to 5. The above calibration was performed separately for ID50 and ID80 readouts. Figure S1 shows the high correlation between readouts for the two assays.

**References**

1. Huang Y, Borisov O, Kee JJ, Carpp LN, Wrin T, Cai S, et al. Calibration of two validated SARS-CoV-2 pseudovirus neutralization assays for COVID-19 vaccine evaluation. Sci Rep. 2021;11(1):23921. Epub 20211214. doi: 10.1038/s41598-021-03154-6. PubMed PMID: 34907214; PubMed Central PMCID: PMCPMC8671391.
