# Supplementary material for: Neutralizing antibody responses over time in a demographically and clinically diverse cohort of individuals recovered from SARS-CoV-2 acquisition in Africa: A cohort study
Source: PLOS Glob Public Health. 2025 Sep 11;5(9):e0005156. doi: 10.1371/journal.pgph.0005156 (PMC12425307; doi:10.1371/journal.pgph.0005156)

**S1 Figure.** Scatterplot of neutralizing antibody (nAb) responses from the VSV assay before and after calibration vs. neutralizing antibody (nAb) responses for the 293T/ACE2 assay, based on the calibration cohort.


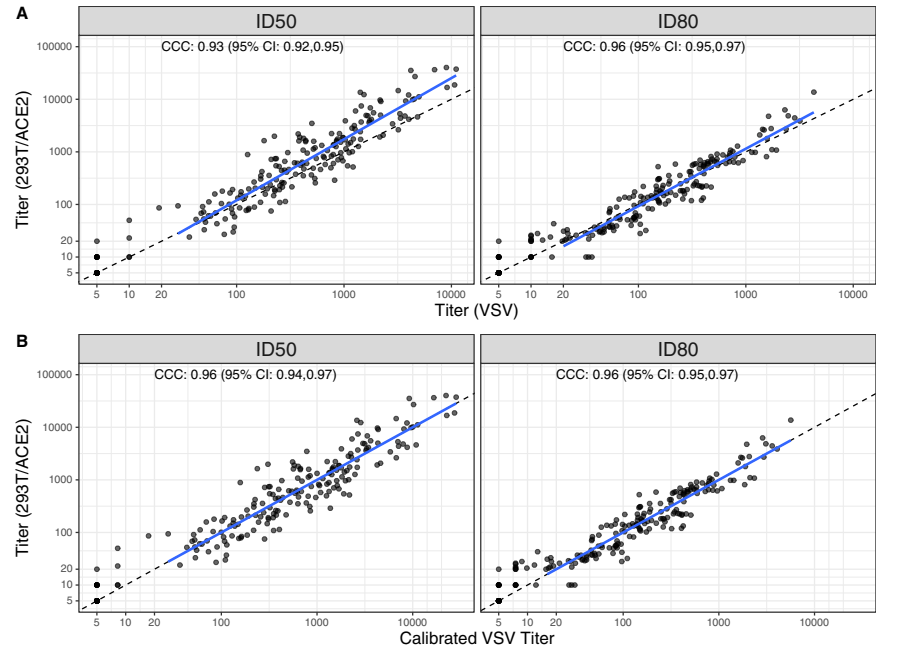

Supplement: S1 Fig — (DOCX) [file pgph.0005156.s004.docx]
