# Supplementary material for: Neutralizing antibody responses over time in a demographically and clinically diverse cohort of individuals recovered from SARS-CoV-2 acquisition in Africa: A cohort study
Source: PLOS Glob Public Health. 2025 Sep 11;5(9):e0005156. doi: 10.1371/journal.pgph.0005156 (PMC12425307; doi:10.1371/journal.pgph.0005156)
Supplement: S1 Table — (DOCX) [file pgph.0005156.s005.docx]

**S1 Table.** Estimated anti-SARS-CoV-2 neutralizing antibody (nAb) response rate and geometric mean (GM) ID50/ID80 titer at enrollment by selected baseline participant characteristics.

| **Characteristics** |  | **n** | **Response Rate (95% CI)** | **Geometric Mean ID50 Titer (95% CI)** | **Geometric Mean ID80 Titer (95% CI)** |
| --- | --- | --- | --- | --- | --- |
| Age category | 18-55 | 265 | 88.30% (83.87%, 91.64%) | 228.0 (175.9, 295.5) | 68.3 (55.3, 84.2) |
|  | >55 | 57 | 100.00% (93.69%, 100.00%) | 719.0 (484.6, 1066.9) | 191.4 (136.1, 269.3) |
| Sex assigned at birth | Female | 205 | 90.24% (85.41%, 93.6%) | 301.6 (228.0, 398.8) | 88.0 (69.7, 111.1) |
|  | Male | 117 | 90.60% (83.95%, 94.67%) | 244.5 (163.7, 365.1) | 72.3 (52.5, 99.5) |
| Region | RSA | 239 | 92.89% (88.91%, 95.51%) | 332.5 (257.8, 428.7) | 91.4 (73.6, 113.4) |
|  | Non-RSA | 84 | 83.33% (73.95%, 89.8%) | 175 (106.7, 287.2) | 61.5 (42.2, 89.5) |
| Living with HIV | No | 283 | 89.75% (85.67%, 92.77%) | 305.7 (238.5, 391.9) | 90.3 (73.8, 110.5) |
|  | Yes | 39 | 94.87% (83.11%, 98.58%) | 145.4 (83.2, 254.0) | 40.6 (25.4, 64.6) |
| BMI | <30 | 170 | 87.65% (81.85%, 91.78%) | 186.7 (133.4, 261.2) | 56.9 (43.5, 74.3) |
|  | ≥30 | 148 | 93.92% (88.85%, 96.77%) | 456.3 (339.7, 613.0) | 127.2 (99.1, 163.3) |
|  | Unknown | 4 | 75% (30.06%, 95.44%) | 101.5 (2.5, 4099.4) | 38.6 (1.8, 824.8) |
| Prolonged viral shedding | No | 317 | 90.54% (86.81%, 93.29%) | 284.7 (226.2, 358.3) | 83.0 (68.7, 100.3) |
|  | Yes | 5 | 80% (37.55%, 96.38%) | 85.6 (4.4, 1666.8) | 35.3 (4.7, 263.9) |
| COVID-19 severity at enrollment | Asymptomatic | 83 | 87.95% (79.22%, 93.32%) | 123.5 (81.0, 188.4) | 38.1 (28.0, 51.9) |
|  | Symptomatic, not hospitalized | 152 | 90.13% (84.36%, 93.93%) | 296.9 (211.6, 416.7) | 85.7 (64.7, 113.4) |
|  | Hospitalized | 87 | 93.10% (85.76%, 96.80%) | 547.5 (362.3, 827.5) | 157.3 (111.2, 222.5) |
| Asthma, COPD, or Emphysema | No | 306 | 90.20% (86.35%, 93.05%) | 273.3 (215.6, 346.3) | 80.5 (66.3, 97.7) |
|  | Yes | 16 | 93.75% (71.67%, 98.89%) | 427.7 (170.9, 1070.9) | 114.8 (53.6, 246.0) |
| Diabetes | No | 277 | 89.53% (85.37%, 92.61%) | 236.0 (183.7, 303.1) | 70.4 (57.5, 86.3) |
|  | Yes | 45 | 95.56% (85.17%, 98.77%) | 790.4 (493.9, 1265.0) | 208.2 (138.7, 312.4) |
| Hypertension | No | 246 | 88.62% (84.04%, 92.01%) | 239.0 (181.9, 314.0) | 69.6 (55.9, 86.8) |
|  | Yes | 76 | 96.05% (89.03%, 98.65%) | 463.3 (313.9, 683.7) | 138.9 (99.7, 193.5) |
| Currently smoke cigarettes or marijuana | No | 291 | 90.38% (86.45%, 93.26%) | 308.4 (242.3, 392.6) | 88.8 (72.8, 108.3) |
|  | Yes | 31 | 90.32% (75.10%, 96.65%) | 110.5 (56.0, 218.0) | 38.6 (23.0, 64.6) |
| Ever smoked cigarettes or marijuana | No | 260 | 91.54% (87.52%, 94.35%) | 338.5 (263.2, 435.4) | 95.8 (77.7, 118.2) |
|  | Yes | 62 | 85.48% (74.66%, 92.17%) | 124.9 (74.7, 209.0) | 42.5 (28.8, 62.7) |
| Time from COVID-19 onset category | <28 days | 24 | 91.67% (74.15%, 97.68%) | 428.8 (163.5, 1124.8) | 110.0 (48.9, 247.3) |
|  | 28-41 days | 68 | 89.71% (80.24%, 94.92%) | 315.3 (180.3, 551.3) | 93.5 (58.4, 149.7) |
|  | 42-55 days | 70 | 84.29% (74.01%, 90.99%) | 197.8 (121.0, 323.2) | 60.9 (41.8, 88.7) |
|  | >56 days | 157 | 92.99% (87.89%, 96.04%) | 277.9 (204.1, 378.4) | 82.2 (63.7, 106.1) |
|  | Unknown | 3 | 100.00% (43.85%, 100.00%) | 2470.4 (80.3, 75956.5) | 342.6 (15.0, 7842.8) |
