# Supplementary material for: Neutralizing antibody responses over time in a demographically and clinically diverse cohort of individuals recovered from SARS-CoV-2 acquisition in Africa: A cohort study
Source: PLOS Glob Public Health. 2025 Sep 11;5(9):e0005156. doi: 10.1371/journal.pgph.0005156 (PMC12425307; doi:10.1371/journal.pgph.0005156)
Supplement: S2 Table — Each predictor of interest is studied independently for its association with the nAb ID80 titer (using log-linear regression). Each regression model adjusts for confounders: COVID-19 severity, age, sex at birth, African region, and days since SARS-CoV-2 diagnosis. (DOCX) [file pgph.0005156.s006.docx]

**S2 Table. Results of multivariate modeling associating baseline participant characteristics with SARS-CoV-2 neutralizing antibody (nAb) ID80 titer at enrollment.** Each predictor of interest is studied independently for its association with the nAb ID80 titer (using log-linear regression). Each regression model adjusts for confounders: COVID­19 severity, age, sex at birth, African region, and days since SARS­CoV­2 diagnosis.

|  | **nAb ID80 titer** | | | |
| --- | --- | --- | --- | --- |
|  | **GMR** | **95% CI** | **P-Value** | **Q-Value^#^** |
| Age (>55 vs. 18-55) | 2.61 | [1.65, 4.14] | <0.001 | <0.001 |
| BMI (≥30 vs <30) | 2.01 | [1.37, 2.94] | <0.001 | 0.001 |
| HIV (Yes vs. No) | 0.33 | [0.19, 0.57] | <0.001 | <0.001 |
| COVID-19 Severity |  |  | <0.001 | <0.001 |
| Symptomatic (not hospitalized) vs. Asymptomatic | 2.41 | [1.56, 3.72] | <0.001 | <0.001 |
| Hospitalized vs. Symptomatic (not hospitalized) | 1.74 | [1.13, 2.69] | 0.013 | 0.020 |
| Hospitalized vs. Asymptomatic | 4.20 | [2.56, 6.88] | <0.001 | <0.001 |
| COPD/Emphysema/Asthma (Yes vs. No) | 1.13 | [0.50, 2.51] | 0.771 | 0.864 |
| Diabetes (Yes vs. No) | 2.08 | [1.24, 3.52] | 0.006 | 0.012 |
| Hypertension (Yes vs. No) | 1.16 | [0.73, 1.84] | 0.530 | 0.652 |
| Cigarettes/Marijuana Smoker (Current) (Yes vs. No) | 0.44 | [0.24, 0.82] | 0.010 | 0.017 |
| Cigarettes/Marijuana Smoker (Ever) (Yes vs. No) | 0.43 | [0.27, 0.69] | <0.001 | 0.001 |
| Days Since SARS-CoV-2 Diagnosis | 1.00 | [0.99, 1.01] | 0.812 | 0.864 |
| Sex Assigned at Birth (Male vs. Female) | 1.03 | [0.71, 1.51] | 0.864 | 0.864 |
| Race (Non-Black vs. Black) | 0.84 | [0.56, 1.25] | 0.380 | 0.507 |

^#^Q-value is false discovery rate (FDR)-adjusted p-value for multiple comparisons.
