# Supplementary material for: Neutralizing antibody responses over time in a demographically and clinically diverse cohort of individuals recovered from SARS-CoV-2 acquisition in Africa: A cohort study
Source: PLOS Glob Public Health. 2025 Sep 11;5(9):e0005156. doi: 10.1371/journal.pgph.0005156 (PMC12425307; doi:10.1371/journal.pgph.0005156)
Supplement: S3 Table — (DOCX) [file pgph.0005156.s007.docx]

**S3 Table.** Estimated anti-SARS-CoV-2 neutralizing antibody (nAb) response rate and geometric mean (GM) ID50 titer by visit among people living with HIV (PLWH) and people without HIV (PWOH) by COVID-19 severity group.

|  | | **Visit 1** | | | **Visit 2** | | | **Visit 3** | | | **Visit 4** | | |
| --- | --- | --- | --- | --- | --- | --- | --- | --- | --- | --- | --- | --- | --- |
| **Severity,**  **HIV Status** | | **N** | **Response Rate**  **(95% CI)** | **GM ID50 Titer**  **(95% CI)** | **N** | **Response Rate**  **(95% CI)** | **GM ID50 Titer**  **(95% CI)** | **N** | **Response Rate**  **(95% CI)** | **GM ID50 Titer**  **(95% CI)** | **N** | **Response Rate**  **(95% CI)** | **GM ID50 Titer**  **(95% CI)** |
| **Asymptomatic** | PLWH | 7 | 100.0%  (64.6%, 100.0%) | 28.3  (8.4, 95.1) | 5 | 80.0%  (37.5%, 96.4%) | 15.0  (4.0, 57.0) | 5 | 80.0%  (37.5%, 96.4%) | 24.0  (1.7, 333.1) | 5 | 80.0%  (37.5%, 96.4%) | 127.2  (3.0, 5443.1) |
|  | PWOH | 76 | 86.8%  (77.4%, 92.7%) | 141.4  (91.0, 219.9) | 53 | 86.8%  (75.2%, 93.4%) | 70.4  (44.2, 112.1) | 52 | 92.3%  (81.8%, 97.0%) | 151.8  (98.7, 233.7) | 46 | 97.8%  (88.7%, 99.6%) | 658.5  (370.1, 1171.6) |
| **Symptomatic,**  **not hospitalized** | PLWH | 13 | 100.0%  (77.2%, 100.0%) | 168.3  (77.8, 363.9) | 10 | 100.0%  (72.2%, 100.0%) | 153.0  (54.4, 430.2) | 10 | 100.0%  (72.2%, 100.0%) | 202.3  (95.4, 428.9) | 9 | 100.0%  (70.1%, 100.0%) | 3014.6  (949.6, 9570.0) |
|  | PWOH | 139 | 89.2%  (83.0%, 93.3%) | 313.1  (217.5, 450.7) | 102 | 85.3%  (77.1%, 90.9%) | 129.6  (82.9, 202.8) | 97 | 88.7%  (80.8%, 93.6%) | 252.4  (156.6, 406.7) | 71 | 97.2%  (90.3%, 99.2%) | 1178.5  (656.1, 2117.0) |
| **Hospitalized** | PLWH | 19 | 89.5%  (68.6%, 97.1%) | 240.3  (100.5, 574.9) | 14 | 100.0%  (78.5%, 100.0%) | 150.1  (55.3, 407.6) | 13 | 92.3%  (66.7%, 98.6%) | 152.5  (47.6, 489.3) | 10 | 100.0%  (72.2%, 100.0%) | 3598.1  (892.5, 14506.0) |
|  | PWOH | 68 | 94.1%  (85.8%, 97.7%) | 689.1  (432.8, 1097.3) | 53 | 96.2%  (87.2%, 99.0%) | 322.0  (200.1, 518.2) | 51 | 94.1%  (84.1%, 98.0%) | 529.7  (325.4, 862.3) | 40 | 97.5%  (87.1%, 99.6%) | 2258.5  (1098.1, 4645.1) |
