# Supplementary material for: Neutralizing antibody responses over time in a demographically and clinically diverse cohort of individuals recovered from SARS-CoV-2 acquisition in Africa: A cohort study
Source: PLOS Glob Public Health. 2025 Sep 11;5(9):e0005156. doi: 10.1371/journal.pgph.0005156 (PMC12425307; doi:10.1371/journal.pgph.0005156)
Supplement: S4 Table — (DOCX) [file pgph.0005156.s008.docx]

**S4 Table.** Estimated anti-SARS-CoV-2 neutralizing antibody (nAb) response rate and geometric mean (GM) ID80 titer by visit among people living with HIV (PLWH) and people without HIV (PWOH) by COVID-19 severity group.

|  | | **Visit 1** | | | **Visit 2** | | | **Visit 3** | | | **Visit 4** | | |
| --- | --- | --- | --- | --- | --- | --- | --- | --- | --- | --- | --- | --- | --- |
| **Severity,**  **HIV Status** | | **N** | **Response Rate**  **(95% CI)** | **GM ID80 Titer**  **(95% CI)** | **N** | **Response Rate**  **(95% CI)** | **GM ID80 Titer**  **(95% CI)** | **N** | **Response Rate**  **(95% CI)** | **GM ID80 Titer**  **(95% CI)** | **N** | **Response Rate**  **(95% CI)** | **GM ID80 Titer**  **(95% CI)** |
| **Asymptomatic** | PLWH | 7 | 100.0%  (64.6%, 100.0%) | 10.7  (5.8, 19.7) | 5 | 80.0%  (37.5%, 96.4%) | 6.9  (5.5, 8.7) | 5 | 80.0%  (37.5%, 96.4%) | 13.5  (1.7, 110.3) | 5 | 80.0%  (37.5%, 96.4%) | 54.0  (3.0, 985.1) |
|  | PWOH | 76 | 86.8%  (77.4%, 92.7%) | 42.9  (31.1, 59.1) | 53 | 86.8%  (75.2%, 93.4%) | 24.7  (18.1, 33.7) | 52 | 92.3%  (81.8%, 97.0%) | 44.7  (31.3, 63.7) | 46 | 97.8%  (88.7%, 99.6%) | 194.3  (115.7, 326.4) |
| **Symptomatic,**  **not hospitalized** | PLWH | 13 | 100.0%  (77.2%, 100.0%) | 40.9  (18.6, 89.8) | 10 | 100.0%  (72.2%, 100%) | 43.4  (15.5, 122.0) | 10 | 100.0%  (72.2%, 100.0%) | 55.1  (25.3, 119.8) | 9 | 100.0%  (70.1%, 100.0%) | 677.2  (233.0, 1967.8) |
|  | PWOH | 139 | 89.2%  (83.0%, 93.3%) | 91.8  (68.2, 123.6) | 102 | 85.3%  (77.1%, 90.9%) | 44.7  (31.3, 63.9) | 97 | 88.7%  (80.8%, 93.6%) | 85.7  (57.7, 127.5) | 71 | 97.2%  (90.3%, 99.2%) | 364.7  (211.2, 630.0) |
| **Hospitalized** | PLWH | 19 | 89.5%  (68.6%, 97.1%) | 65.9  (32.6, 133.6) | 14 | 100.0%  (78.5%, 100.0%) | 47.4  (20.8, 107.6) | 13 | 92.3%  (66.7%, 98.6%) | 50.7  (20.2, 127.2) | 10 | 100.0%  (72.2%, 100.0%) | 809.3  (230.9, 2836.9) |
|  | PWOH | 68 | 94.1%  (85.8%, 97.7%) | 200.5  (136.3, 295.2) | 53 | 96.2%  (87.2%, 99.0%) | 100.1  (67.2, 149.2) | 51 | 94.1%  (84.1%, 98.0%) | 165.5  (107.8, 254.1) | 40 | 97.5%  (87.1%, 99.6%) | 666.2  (344.4, 1288.7) |
