# Supplementary material for: Neutralizing antibody responses over time in a demographically and clinically diverse cohort of individuals recovered from SARS-CoV-2 acquisition in Africa: A cohort study
Source: PLOS Glob Public Health. 2025 Sep 11;5(9):e0005156. doi: 10.1371/journal.pgph.0005156 (PMC12425307; doi:10.1371/journal.pgph.0005156)
Supplement: Attachment — Submitted filename: Response to Reviewers.docx [file pgph.0005156.s010.docx]

1. Does this manuscript meet PLOS Global Public Health’s [publication criteria](https://journals.plos.org/globalpublichealth/s/criteria-for-publication)? Is the manuscript technically sound, and do the data support the conclusions? The manuscript must describe methodologically and ethically rigorous research with conclusions that are appropriately drawn based on the data presented.

Reviewer #1: Yes

2. Has the statistical analysis been performed appropriately and rigorously?

Reviewer #1: Yes

3. Have the authors made all data underlying the findings in their manuscript fully available (please refer to the Data Availability Statement at the start of the manuscript PDF file)?

The [PLOS Data policy](https://journals.plos.org/globalpublichealth/s/data-availability) requires authors to make all data underlying the findings described in their manuscript fully available without restriction, with rare exception. The data should be provided as part of the manuscript or its supporting information, or deposited to a public repository. For example, in addition to summary statistics, the data points behind means, medians and variance measures should be available. If there are restrictions on publicly sharing data—e.g. participant privacy or use of data from a third party—those must be specified.

Reviewer #1: Yes

4. Is the manuscript presented in an intelligible fashion and written in standard English?

PLOS Global Public Health does not copyedit accepted manuscripts, so the language in submitted articles must be clear, correct, and unambiguous. Any typographical or grammatical errors should be corrected at revision, so please note any specific errors here.

Reviewer #1: Yes

5. Review Comments to the Author

Please use the space provided to explain your answers to the questions above. You may also include additional comments for the author, including concerns about dual publication, research ethics, or publication ethics. (Please upload your review as an attachment if it exceeds 20,000 characters)

Reviewer #1: This is a thorough and well conducted study of nAb response in Africa during the initial stages of the covid-19 pandemic.


The increase in antibody response over visits 3 and 4 must be due to vaccination and infection. Is it possible to do an analysis where you look at trajectories where values prior to vaccination/infection are censored? You could also look at the effect of vaccination/infection on boosting nAbs. If you can’t do such analyses, you should list this as a limitation.

**Response**: Thank you to the reviewer for this comment. We have added the following to the manuscript at or around line 400 (added text in bold underline in this response):

“Data regarding post-enrollment SARS-CoV-2 reacquisition was rarely collected due to limited accessibility of SARS-CoV-2 testing kits and policies limiting the type of allowable research clinic procedures early in the pandemic**, and vaccine access and reporting was inconsistent during the study period. These limitations impaired our ability to precisely characterize the impacts of reinfection and vaccination on neutralizing antibody trajectories, particularly through the last two study visits that took place during or after the Beta and Delta waves and the gradual introduction of vaccines.**”

The manuscript also addresses regional vaccination introductions and SARS-CoV-2 variant waves leading to increased infections or re-infections, as well as the study’s limitations related to these events, in lines 307-308, 353-364, and 380-385. Furthermore, while we do not have the necessary data quality to support formally “censoring” based on vaccine or (re)infection status, in these earlier sections of the manuscript we describe the neutralizing antibody trajectory through the first two visits, before the advent of vaccines and significant viral variant waves.

6. PLOS authors have the option to publish the peer review history of their article ([what does this mean?](https://journals.plos.org/globalpublichealth/s/editorial-and-peer-review-process#loc-peer-review-history)). If published, this will include your full peer review and any attached files.


**Do you want your identity to be public for this peer review?** If you choose “no”, your identity will remain anonymous but your review may still be made public.


For information about this choice, including consent withdrawal, please see our [Privacy Policy](https://www.plos.org/privacy-policy).

Reviewer #1: No

**Comments to the Reviewer**: We thank the reviewer for their time and for providing kind feedback and comments. We have addressed comment #5.
